# Ferroptosis: a new strategy for Chinese herbal medicine treatment of diabetic nephropathy

**DOI:** 10.3389/fendo.2023.1188003

**Published:** 2023-06-09

**Authors:** Maoying Wei, Xingxing Liu, Zhijuan Tan, Xiaochan Tian, Mingdi Li, Junping Wei

**Affiliations:** ^1^ Department of Endocrinology, Guang’anmen Hospital, China Academy of Chinese Medical Sciences, Beijing, China; ^2^ Department of Emergency, Guang’anmen Hospital, China Academy of Chinese Medical Sciences, Beijing, China; ^3^ Department of Traditional Chinese Medicine, The Seventh Hospital of Xingtai, Xingtai, Heibei, China

**Keywords:** diabetic nephropathy, ferroptosis, Chinese herbal medicine, renal protection, mechanism

## Abstract

Diabetic nephropathy (DN) is a serious microvascular complication of diabetes. It has become a leading cause of death in patients with diabetes and end-stage renal disease. Ferroptosis is a newly discovered pattern of programmed cell death. Its main manifestation is the excessive accumulation of intracellular iron ion-dependent lipid peroxides. Recent studies have shown that ferroptosis is an important driving factor in the onset and development of DN. Ferroptosis is closely associated with renal intrinsic cell (including renal tubular epithelial cells, podocytes, and mesangial cells) damage in diabetes. Chinese herbal medicine is widely used in the treatment of DN, with a long history and definite curative effect. Accumulating evidence suggests that Chinese herbal medicine can modulate ferroptosis in renal intrinsic cells and show great potential for improving DN. In this review, we outline the key regulators and pathways of ferroptosis in DN and summarize the herbs, mainly monomers and extracts, that target the inhibition of ferroptosis.

## 1 Introduction

Diabetes mellitus is one of the major diseases that seriously endanger human health. According to the International Diabetes Federation, 536.6 million adults aged 20 to 79 had diabetes mellitus worldwide in 2021, and that number is expected to rise to 783.2 million by 2045 (1). Diabetic nephropathy (DN) is one of the major microvascular complications of diabetes mellitus and the most common cause of end-stage renal disease (2). According to statistics, DN occurs in 20% to 40% of patients with type 1 and type 2 diabetes mellitus (3). About 30% to 50% of end-stage renal disease worldwide is caused by DN (4). Previous studies have shown that hyperglycemia, hypertension, dyslipidemia, and being overweight or obese are common risk factors for DN (5). Therefore, lifestyle changes, aggressive glycemic control, blood pressure reduction, lipid regulation, and reduction of proteinuria are currently the main measures in the clinical management of DN. In the past twenty years, angiotensin-converting enzyme inhibitors (ACEI) and angiotensin receptor blockers (ARB) have shown good renoprotective effects in patients with DN. In recent years, newer drugs, including sodium-glucose co-transporter 2 inhibitors, glucagon-like peptide-1 receptor agonists, mineralocorticoid receptor antagonists, and endothelin antagonists, have also provided more options for the treatment of DN (6). However, some DN patients still progress to end-stage renal disease. Hence, finding new targets for the prevention and treatment of DN has become an urgent problem in this field.

The pathogenesis of DN has not been fully elucidated. Various pathogenic mechanisms such as oxidative stress (7), inflammatory response (8), the accumulation of advanced glycation end products (9, 10), endoplasmic reticulum stress (11), autophagy (12), and pyroptosis (13) are involved in the pathological process of DN. Ferroptosis is a newly discovered iron-dependent novel form of programmed cell death that differs morphologically, biochemically, and genetically from apoptosis, autophagy, and necrosis (14). The occurrence of ferroptosis in cells is usually accompanied by a large accumulation of iron and lipid peroxidation (15). Increased iron accumulation, free radical production, fatty acid supply, and lipid peroxidation are currently thought to be key to the induction of ferroptosis. Previous studies have shown that renal iron overload is prevalent in diabetic animal models, and even serum creatinine and urine protein levels are positively correlated with renal iron and ferritin levels (16, 17). Excess iron not only increased oxidative/nitrative stress and decreased antioxidant capacity but also activated the renin-angiotensin system, thereby promoting the progression of DN (17, 18). Since the presence of ferroptosis in DN was first reported by Wang et al. (19) in 2020. With the advancement of research, accumulating evidence suggests that ferroptosis is an important factor in promoting the occurrence and development of DN (20). Therefore, targeted inhibition of ferroptosis is expected to be a new tool for the treatment of DN. In recent years, Chinese herbal medicine has played an increasingly important role in the prevention and treatment of DN. Several clinical studies have shown that either the use of Chinese herbal medicine alone or in combination with ACEI or ARB helps to improve renal function and reduce proteinuria in patients with DN (21–23). More importantly, there is a growing body of research on Chinese herbal medicine to prevent and treat DN by regulating ferroptosis. This paper reviews the key regulators and signaling pathways of ferroptosis in DN and the research progress of Chinese herbal medicine in the treatment of DN by regulating ferroptosis, in order to provide a new research direction for the treatment of DN.

## 2 Key regulators and signaling pathways of ferroptosis in diabetic nephropathy

**Figure 1:**
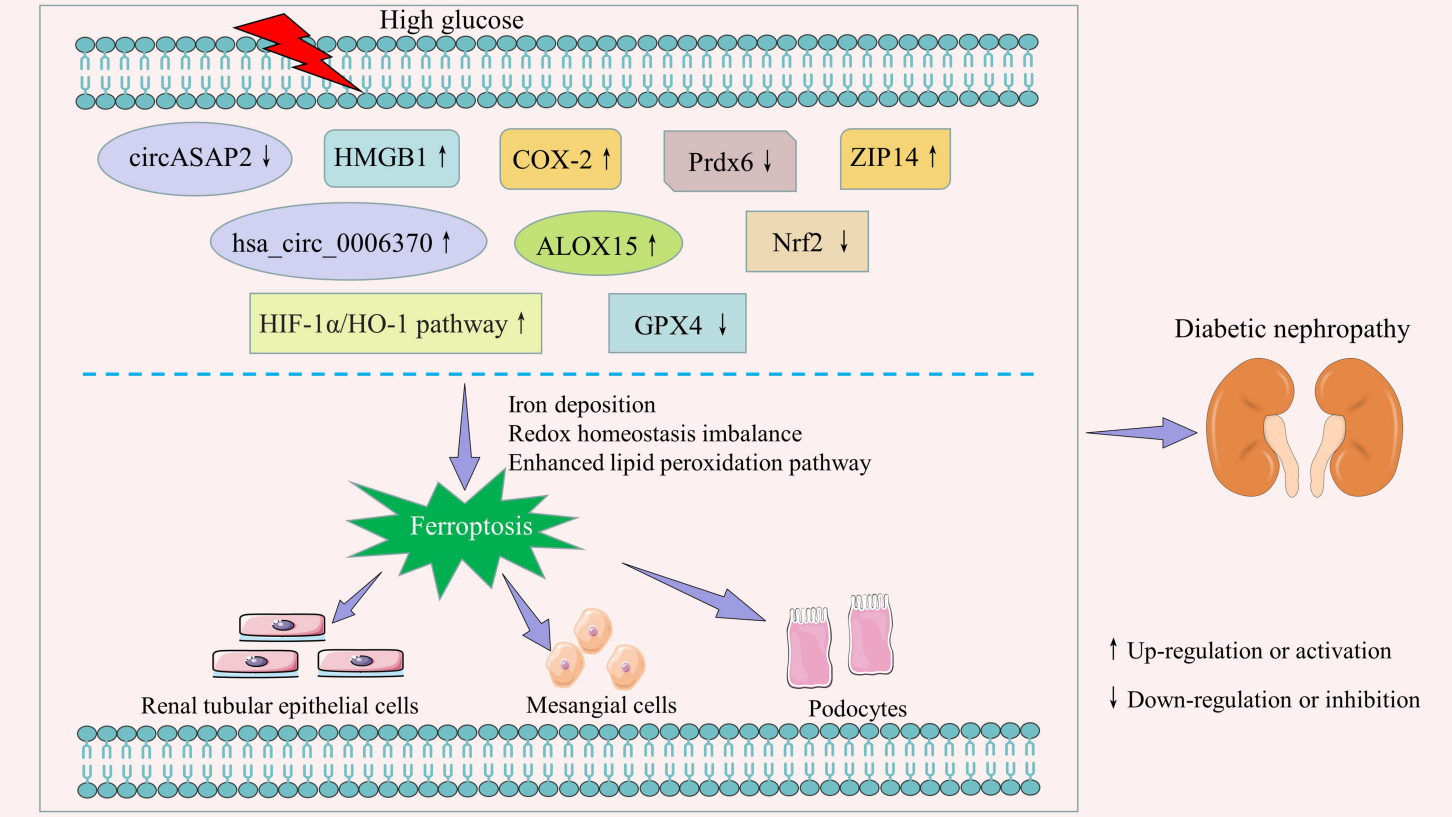
Key regulators and signaling pathways of ferroptosis in diabetic nephropathy.

Ferroptosis in renal intrinsic cells is prevalent in the pathological process of DN. Previous studies have demonstrated that it is regulated by a variety of regulatory factors and signal transduction pathways (**Figure 1**; **Supplementary Table 1**). We have summarized these in detail.

### 2.1 Non-coding RNAs

It is well known that although non-coding RNAs are not directly involved in the protein translation process, they can produce non-coding transcripts that regulate gene expression and protein function (24). The most studied non-coding RNAs are microRNA, long non-coding RNA, and circular RNA. Previous studies have found that non-coding RNAs are widely involved in the biological processes of renal intrinsic cells, including cell proliferation (25), apoptosis (26), cellular senescence (27, 28), autophagy (29, 30), and pyroptosis (31, 32). Clinical data have confirmed that non-coding RNAs are important biomarkers for the early diagnosis and prognosis of patients with diabetic kidney disease (33, 34). The emerging evidence has shown that non-coding RNAs are important regulators of ferroptosis (35, 36). Li et al. (37) found that circASAP2 expression was downregulated and miR-770-5p expression was upregulated in DN mouse serum, kidney, and high-glucose cultured HK-2 cells. Upregulation of miR-770-5p suppressed the target gene SOX2 and its induced SLC7A11 expression, thereby promoting inflammation and ferroptosis. Xiong et al. (38) observed that hsa_circ_0006370 expression was significantly upregulated in HK-2 cells cultured with high glucose, while interference with hsa_circ_0006370 expression significantly increased the survival of HK-2 cells. Mechanistically, hsa_circ_0006370 silencing attenuated ferroptosis by inhibiting intracellular reactive oxygen species (ROS) production, enhancing antioxidant capacity, reducing iron ion levels, and regulating ferroptosis-associated protein expression. Therefore, circASAP2 and hsa_circ_0006370 may be potential intervention targets for DN ferroptosis.

### 2.2 Cyclooxygenase-2

Cyclooxygenase (COX), also known as prostaglandin synthase, is the key rate-limiting enzyme that catalyzes the conversion of arachidonic acid to prostaglandins. At least three isoforms of COX have been identified, namely COX-1, COX-2, and COX-3 (39). COX-3 is poorly studied, and COX-1 is constitutively expressed in most tissues and cells (40). COX-2 is an inducible enzyme that is less expressed in normal tissues. However, COX-2 is abundantly expressed in certain inflammatory stimuli, such as cytokines, tumor inducers, oncogenes, etc. (41, 42). In recent years, numerous studies have confirmed that COX-2 expression is significantly increased in mesangial cells, podocytes, and renal tubular epithelial cells induced by high glucose, as well as in the kidney of animal models of diabetes (43–47). Wei et al. (43) pointed out that activation of the c-Src/NF-κB/COX-2 pathway in the high-glucose state promoted extracellular matrix accumulation and mesangial proliferation, which exacerbated glomerulosclerosis. Ma et al. (48) found a positive correlation between COX-2 expression levels and low-density lipoprotein receptor expression levels in the kidney of diabetic rats. Increased COX-2 expression exacerbated renal lipid accumulation and inflammatory cytokine production, including interleukin-1beta (IL-1β), interleukin 6 (IL-6), tumor necrosis factor alpha (TNF-α), and monocyte chemoattractant protein-1 (MCP-1). In addition, COX-2 also contributes to diabetic nephropathy through the glomerular EP4 receptor (49). The latest report showed that increased COX-2 expression was accompanied by ferroptosis, while COX-2 knockdown reduced the sensitivity of renal tubular cells to ferroptosis in the high glucose state (47).

### 2.3 Arachidonate 15-lipoxygenase

Lipoxygenases are non heme-iron containing dioxygenases, and arachidonate 15-lipoxygenase (ALOX15) is a member of its family (50). Several studies have shown that ALOX15 is a key mediator in the formation of lipid peroxidation products and ultimately leads to ferroptosis (51, 52). In tert-butyl hydroperoxide-induced primary cortical neurons, Zhao et al. (53) observed that ALOX15 inhibition reversed SSAT1 upregulation-triggered neuronal loss and ferroptosis. Similarly, Gao et al. (54) found that inhibition of lipid peroxidation by downregulating the expression of ALOX15 contributed to the attenuation of ferroptosis in microglia and endothelial cells after subarachnoid hemorrhage. In addition, both the selective ALOX15 inhibitor PD146176 and siRNA-mediated silencing of ALOX15 significantly reduced ferroptosis in erastin-induced and RSL3-induced cancer cells (e.g., HT1080, Calu-1). Conversely, ALOX15 activators enhanced the sensitivity of cancer cells to these compounds (52). In recent years, ALOX15 has been extensively studied in diabetes and its complications (55–58). In the field of DN, Kim et al. (59) found that the 12/15-lipoxygenase and TGF-β pathways can cross-talk and activate each other. Another study showed that 12/15-lipoxygenase knockout reduced the expression of SET7 and profibrotic genes, which in turn improved proteinuria and renal pathological changes in diabetic mice (60). It was recently reported that ALOX15 protein expression was significantly increased in the renal tissues of streptozotocin (STZ)-induced diabetic mice, *db/db* mice, and DN patients. Correlation analysis suggested that ALOX15 protein expression was closely associated with renal pathological changes in DN patients, such as glomerular lesions, interstitial fibrosis and tubular atrophy, and interstitial inflammation. More importantly, immunofluorescence double staining also observed glutathione peroxidase 4 (GPX4) and ALOX15 expression in the same region. This study confirmed that ALOX15 is an important factor involved in DN ferroptosis (58). Therefore, targeting ALOX15 provides a new idea for the intervention of DN ferroptosis.

### 2.4 Peroxiredoxin 6

Peroxidases (Prdxs) are a family of antioxidant proteins widely found in prokaryotes and eukaryotes. Peroxidase 6 (Prdx6) is the only member of the Prdxs family that belongs to the 1-cysteine subclass. Unlike other Prdxs, Prdx6 is a bifunctional enzyme with both glutathione peroxidase and phospholipase A2 activities (61). It has been found that Prdx6 is widely expressed in human and mouse kidneys. Moreover, Prdx6 is involved in the maintenance of PH homeostasis by interacting with anion exchanger 1 (62). Another study showed that Prdx6 overexpression attenuated lipopolysaccharide-induced apoptosis in primary renal proximal tubule cells by scavenging ROS (63). Bioinformatics analysis showed that seven hub ferroptosis-related genes, including Prdx6, could accurately distinguish between diabetic kidney disease and control samples (64). Zhang et al. (65) found that Prdx6 was significantly downregulated in high glucose-induced podocytes and in the kidneys of DN mice. Overexpression of Prdx6 *in vivo* reduced urinary protein, blood creatinine, and urea nitrogen in DN mice. *In vitro*, Prdx6 overexpression ameliorated podocyte dysfunction by attenuating high glucose-induced oxidative stress and ferroptosis. Therefore, enhancing the activity of Prdx6 is an important way to alleviate ferroptosis in high glucose-induced podocytes.

### 2.5 ZRT/IRT-like protein 14

ZRT/IRT-like protein 14 (ZIP14, also known as SLC39A14), a member of the solute-linked carrier 39 family, is a transporter protein that mediates cellular uptake of zinc (66), manganese (67), and iron (68, 69). As an essential trace element, zinc and its carrier protein are closely related to the synthesis, secretion, and biological activity of insulin (70). Previous studies have shown the presence of ZIP14 in pancreatic β-cells, and modulation of ZIP14 activity is expected to be an important target for improving β-cell dysfunction (71). Lawson et al. (72) discovered that prolonged high glucose stimulation disrupted the expression of several ZIP transporter proteins, including SLC39A6, SLC39A7, and SLC39A14, depleted zinc from pancreatic β-cells, and resulted in the loss of β-cell markers, thereby promoting β-cell dedifferentiation. Maxel et al. (71) demonstrated that silencing of ZIP14 in pancreatic β-cells affects insulin processing and disturbs mitochondrial function. Recent studies have found that ZIP14 can be detected in both the proximal and distal tubules of the human kidney (73). In human proximal tubule epithelial cells, ZIP14 is involved not only in non-transferrin-bound iron uptake but also in transferrin-bound iron-derived iron uptake (74). Tubular iron deposition is common in chronic kidney disease, with glomerular dysfunction as the primary pathology, accompanied by increased ZIP14 expression in the proximal and distal tubules (73). Iron overload is both a major source of ROS and a key participant in ferroptosis (75). The emerging evidence has shown that ZIP14 expression was upregulated in the renal tubules of both DN patients and DN rats, as well as in HK-2 cells cultured with high glucose. Increased ZIP14 promotes iron deposition and is involved in diabetic kidney injury *via* ferroptosis (76). This study confirms a novel role of ZIP14 in ferroptosis in DN. Thus, ZIP14 may become an important pharmacological target for the treatment of DN.

### 2.6 High mobility group box 1

High mobility group box 1 (HMGB1), a highly conserved nuclear protein, is widely distributed in mammalian cells. As a DNA binding protein, nuclear HMBG1 is involved in a series of key nuclear events, such as nucleosome stability and sliding, gene transcription, DNA replication, DNA repair, gene transfer, gene delivery, etc. (77–79). Extracellularly, HMGB1 acts as a damage-associated molecular pattern molecule involved in cell differentiation, inflammation, immunity, cell migration, tissue regeneration, and other biological processes (77, 79, 80). Additionally, cytoplasmic HMBG1 is important for autophagy regulation (81). DN is a chronic inflammatory disease in which low-grade inflammation and the immune response are important mechanisms in its pathogenesis (82–85). HMGB1, an important mediator that can participate in the inflammatory response, drives the development of DN. Clinical data have shown that serum HMGB1 is closely associated with urinary albumin excretion and renal tubular damage in patients with type 2 diabetes (86). Preclinical studies have demonstrated that HMGB1 promotes the expression of markers of oxidative stress, inflammation, and fibrosis in DN cell models (87, 88). Knockdown of HMGB1 or inhibition of HMGB1-mediated inflammatory signaling pathways (e.g., HMGB1-RAGE-NF-κB and HMGB1-TLR4) significantly attenuated podocyte apoptosis, epithelial mesenchymal transition, renal inflammation, and fibrotic processes (87, 89, 90). Recent studies have revealed that HMGB1 is also an important regulator of ferroptosis (91, 92). Both type I and type II ferroptosis activators, including erastin, sorafenib, RSL3, and FIN56, induced the release of HMGB1 (93). HMGB1 could regulate ferroptosis through the RAS-JNK/p38 pathway (94). Zhao et al. (95) found that cytoplasmic HMGB1 induced renal tubular ferroptosis through regulation of acyl-CoA synthetase long-chain family member 4 (ACSL4). In HL-60/NRASQ61L cells, Ye et al. (94) observed that knockdown of HMGB1 inhibited erastin-induced ROS and malondialdehyde (MDA) production, degradation of GPX4, and cell death in an iron-mediated lysosomal pathway. It has been recently reported that serum HMGB1 expression is elevated in DN patients compared to healthy controls, accompanied by an increase in ferroptosis. Further studies revealed that knockdown of HMGB1 significantly inhibited high glucose-induced ferroptosis in mesangial cells. Mechanistically, silencing of HMGB1 not only decreased the expression of ACSL4, prostaglandin-endoperoxide synthase 2 (PTGS2), and NADPH oxidase 1 (NOX1), but also increased the level of GPX4 (96). This study suggests that HMGB1 inhibition may have potential therapeutic value for ferroptosis-associated DN.

### 2.7 Nuclear factor erythroid 2-related factor 2

Nuclear factor erythroid 2-related factor 2 (Nrf2) is a key transcription factor in the body’s antioxidant stress response. In addition to antioxidant responses, Nrf2 is involved in the regulation of a variety of other biological processes, including drug detoxification, amino acid metabolism, lipid metabolism, heme and iron metabolism, autophagy, unfolded protein response, inflammation, and immunity (97). Several studies have found significantly lower circulating Nrf2 levels in DN patients compared to healthy controls (96, 98). Nrf2 deficiency resulted in more severe diabetic symptoms, renal inflammation, and interstitial renal fibrosis in Akita diabetic model mice (99). Conversely, Nrf2 activation delayed DN progression by increasing the expression of the downstream antioxidant enzymes HO-1 and NADPH quinone oxidoreductase-1 (NQO-1) (100, 101). Ferroptosis is a new regulatory cell death mode. Three key events are required for its occurrence: iron deposition, lipid peroxidation, and glutathione depletion (102). Accumulating studies confirm that Nrf2 is an important regulator of ferroptosis (103, 104). Many components that are involved in the prevention of lipid peroxidation and the regulation of cellular iron homeostasis are target genes of Nrf2 (105–107). New evidence suggests that specific knockdown of Nrf2 enhanced the susceptibility of HK-2 cells to ferroptosis under high glucose conditions, while upregulation of Nrf2 expression contributed to the inhibition of ferroptosis and thus reduced kidney injury in diabetic mice (108). Another study found that high glucose promoted salusin-β expression in HK-2 cells in a time- and dose-dependent manner. Upregulated salusin-β mediated ferroptosis in renal tubular epithelial cells by inhibiting the Nrf2 expression (109). Taken together, the above results demonstrate that Nrf2 is an important regulator of ferroptosis in DN.

### 2.8 GPX4

GPX4 is a unique antioxidant enzyme in mammals. Functionally, GPX4 converts lipid hydroperoxides to non-toxic lipid alcohols. This limits the propagation of lipid peroxidation in the membrane (110). GPX4 is a key and central regulator of ferroptosis, as is now generally accepted (111, 112). Overexpressing and knocking down GPX4 modulates the lethality of 12 ferroptosis inducers (113). In addition, GPX4 is closely associated with kidney damage. Inducible GPX4 disruption was reported to cause acute renal failure and death in mice as early as 2014 (114). In recent years, GPX4 has gradually become the star molecule in the DN field. A number of studies have shown that the expression of GPX4 is significantly lower in kidney biopsy samples from patients with diabetic kidney disease (115–117). Moreover, reduced GPX4 levels were associated with the severity of diabetic kidney disease and an increased risk of progression to end-stage renal disease (117). A recent study found that activation of the SIRT3-SOD2-GPX4 signaling pathway, including upregulation of sirtuin 3 (SIRT3) expression, inhibition of superoxide dismutase 2 (SOD2) acetylation, and consequently promotion of GPX4 expression, helped restore mitochondrial redox homeostasis, thereby alleviating ferroptosis in DN models (118).

### 2.9 HIF-1α/HO-1 pathway

Hypoxia-inducible factor-1alpha (HIF-1α) is an important regulator of cellular oxygen homeostasis. Previous studies have found that HIF-1α is aberrantly expressed in the serum of diabetic patients and in the kidneys of DN patients and is significantly associated with the progression of interstitial renal fibrosis (119–121). HIF-1α, as a transcription factor, is able to regulate the expression of its downstream target, heme oxygenase-1 (HO-1) (122). It has been reported that deletion of HIF-1α in proximal renal tubule cells exacerbates mitochondrial dysfunction and renal injury in diabetic mice *via* the HO-1 pathway (123). A number of studies have also shown that the HIF-1α/HO-1 signaling pathway plays an important role in the regulation of ferroptosis (124–126). Destabilization of HIF-1α enhanced the sensitivity of gastric cancer cells to erastin and RSL3 (127). Furthermore, HO-1 regulates ROS production and iron metabolism (128). HO-1 deficiency promotes erastin-induced ferroptosis (129). Another study showed that HO-1 in renal proximal tubule cells is similarly antiferroptotic (130). Diabetes promoted renal ROS formation, lipid peroxidation, and renal tubular iron overload. However, this phenomenon can be blocked by the ferroptosis inhibitor Ferrostatin-1. Further studies revealed that ferroptosis may exacerbate proteinuria, tubular injury, and renal fibrosis in *db/db* mice *via* the HIF-1α/HO-1 pathway (131). Therefore, targeting the HIF-1α/HO-1 signaling pathway and regulating ferroptosis may be an effective strategy for improving DN.

The above findings suggest that ferroptosis is closely associated with renal intrinsic cell death in diabetic conditions, mainly in renal tubular epithelial cells, mesangial cells, and podocytes. Non-coding RNAs (e.g., circASAP2, hsa_circ_0006370), COX-2, ALOX15, Prdx6, etc. are important regulators of ferroptosis in renal cells. HIF-1α/HO-1 is a critical regulatory pathway. Therefore, an in-depth exploration of the role of these regulators and signaling pathways in ferroptosis is important for both understanding the pathogenesis of DN and finding new drug targets.

## 3 Modulatory role of Chinese herbal medicine in ferroptosis in diabetic nephropathy

**Figure 2:**
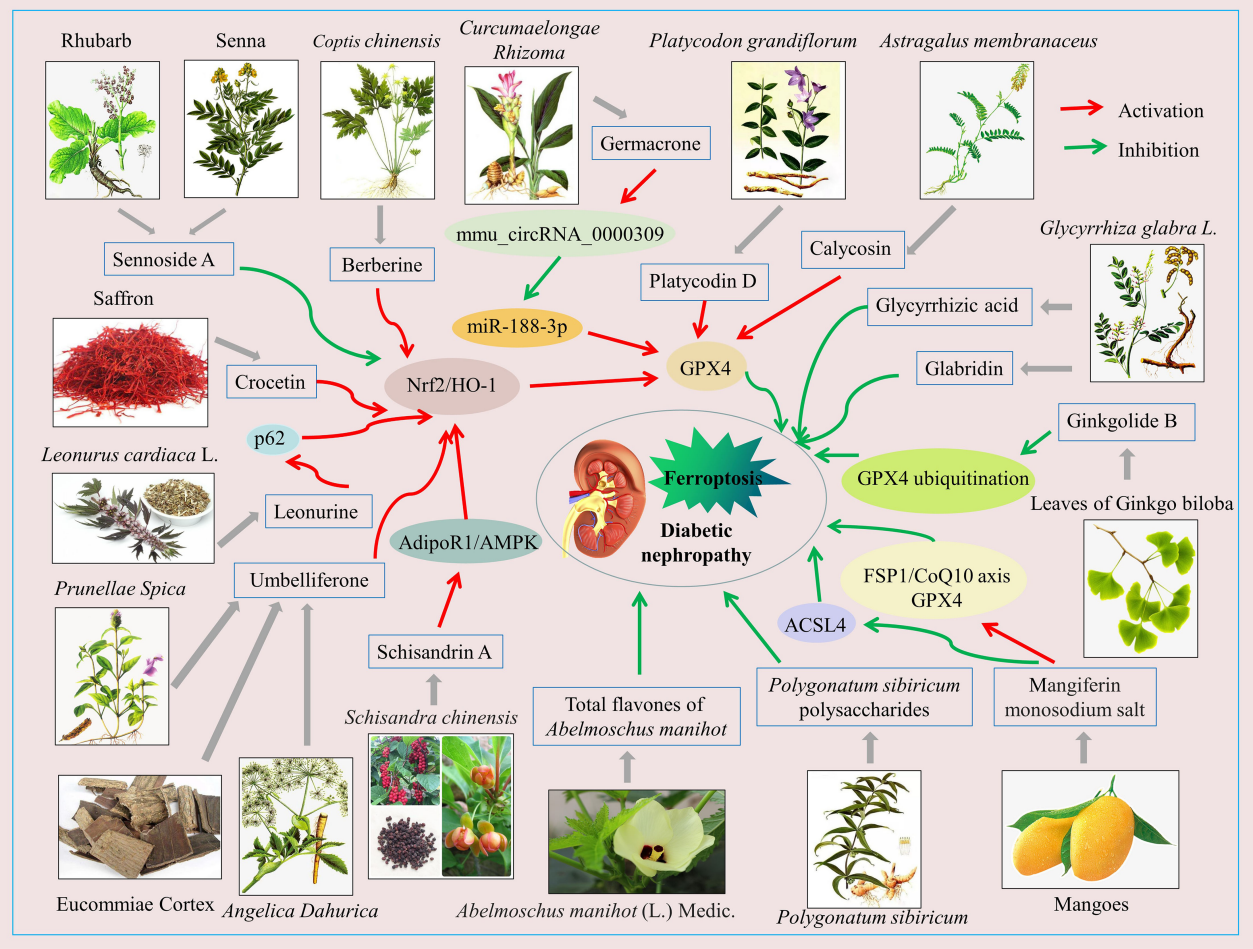
Schematic of Chinese herbal medicine against diabetic nephropathy by inhibiting ferroptosis. The activation of Nrf2/HO-1, GPX4, FSP1/CoQ10, AdipoR1/AMPK signaling pathways and so on, or the inhibition of the ASCL4 signaling pathway, can inhibit ferroptosis in renal intrinsic cells, which benefits diabetic nephropathy treatment.

**Figure 3:**
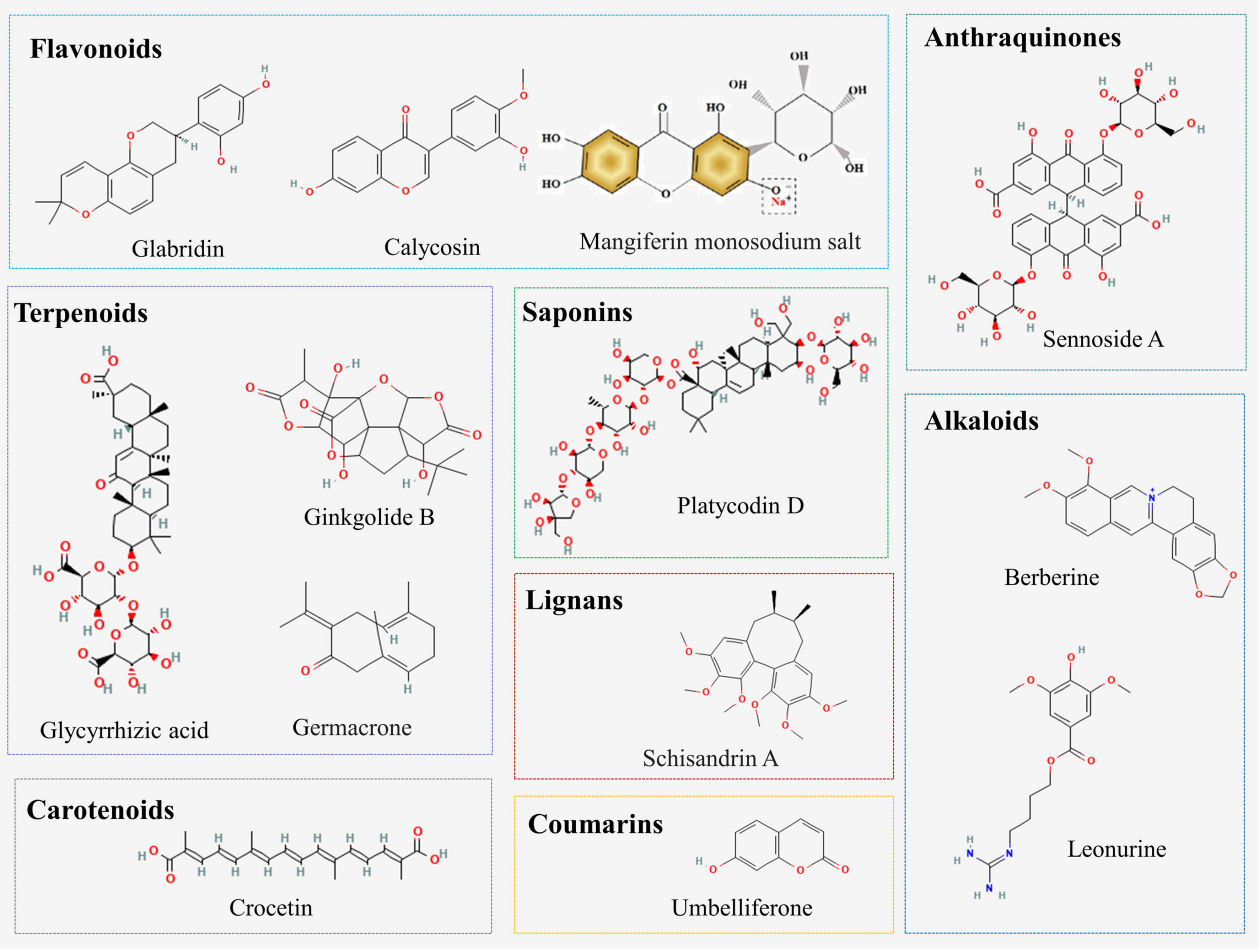
Chemical structures of natural compounds from Chinese herbal medicine.

A variety of natural compounds and extracts from Chinese herbal medicine have been reported to delay the progression of DN by inhibiting ferroptosis (**Figure 2**; **Supplementary Table 2**). Common natural compounds include flavonoids, terpenoids, anthraquinones, alkaloids, etc. (**Figure 3**). We will comprehensively review the anti-ferroptotic properties of Chinese herbal medicine and its potential in the treatment of DN.

### 3.1 Flavonoids

#### 3.1.1 Glabridin

Glabridin is a prenylated isoflavone extracted from the roots and rhizomes of *Glycyrrhiza glabra L* (132). Previous studies have found that glabridin has hepatoprotective, neuroprotective, antidiabetic, and antioxidant effects in animal models of diabetes (133–135). Tan et al. (136) found that glabridin not only reversed hyperglycemia and insulin resistance in diabetic rats but also attenuated diabetes-induced damage to kidney function and structure. Mechanistically, glabridin increased SOD, glutathione (GSH), and catalase (CAT) activities; up-regulated GPX4, SLC7A11, and SLC3A2 expression; down-regulated transferrin receptor 1 (TfR1), vascular endothelial growth factor (VEGF), p-AKT, and p-ERK1/2 expression; and decreased MDA content and iron concentration *in vitro* and *in vivo*. These results suggest that glabridin delays DN progression in association with inhibition of oxidative stress, ferroptosis, and the VEGF/Akt/ERK signaling pathway.

#### 3.1.2 Calycosin

Calycosin is an isoflavone phytoestrogen extracted from the Chinese herb *Astragalus membranaceus*, which has various pharmacological activities such as anti-inflammatory, antioxidant, anti-diabetic, anti-osteoporosis, anti-tumor, hepatoprotective, neuroprotective, and cardioprotective (137). In recent years, an increasing number of studies have explored the role and underlying mechanisms of calycosin in the treatment of DN (138–140). Huang et al. (141) observed that calycosin improved renal function, attenuated tubular injury, inhibited renal fibrosis in db/db mice *in vivo*, and increased the viability of high glucose-induced HK-2 cells *in vitro*. This protective effect of calycosin was associated with its reduction of lactate dehydrogenase (LDH), MDA, and lipid ROS levels; increase of GSH activity; promotion of GPX4 expression; and inhibition of nuclear receptor coactivator 4 (NCOA4) expression in DN models. Moreover, the above effects of calycosin were reversed when co-treated with the ferroptosis inducer erastin. These results suggest that calycosin reduces diabetes-induced kidney injury by inhibiting ferroptosis.

#### 3.1.3 Total flavones of *Abelmoschus manihot*

Total flavones of *Abelmoschus manihot* (TFA) is a total flavonoid component extracted from the flowers of *Abelmoschus manihot* (L.) Medic. There are eight flavone glycosides whose chemical structures have been identified in TFA, including quercetin-3-O-robinobioside, hyperoside, quercetin, myricetin, quercetin-3′-O-glucoside, gossypetin, gossypetin-3-O-glucoside, and isoquercetin (142). Previous studies have shown that TFA has significant anti-inflammatory, endoplasmic reticulum stress-inhibiting, and podocyte-protective activities and is therefore widely used in the treatment of inflammatory bowel disease, DN, and chronic renal failure (142–146). Recent studies have found that TFA has effects similar to those of ferrostatin-1, a ferroptosis inhibitor. TFA inhibited ROS production, upregulated GPX4 and SLC7A11 expression, downregulated TfR1 and ACSL4 expression, and decreased MDA levels and iron content in the DN model (147). These data suggest that inhibition of ferroptosis in renal tubular cells by reducing iron deposition and improving antioxidant capacity may be an important mechanism by which TFA attenuates diabetic tubulopathy.

#### 3.1.4 Mangiferin monosodium salt

Mangiferin is a natural flavonoid extracted mainly from mangoes and has a wide range of pharmacological activities, including antioxidant (148), anti-inflammatory and immunomodulatory (149), anti-diabetic (150), anti-cancer (151), anti-fibrotic (152), nephroprotective (153), cardioprotective (154), and neuroprotective (155). However, the poor solubility and low bioavailability of mangiferin limit its wide application (156). The mangiferin monosodium salt (MGM) obtained by structural modification of mangiferin can improve the solubility and bioavailability of mangiferin to some extent (157). The emerging evidence has shown that MGM also has a good renoprotective effect in DN rats. Mechanistically, MGM not only attenuated systemic insulin resistance (IR)-induced renal inflammation by inhibiting the MAPK/NF-κB signaling axis, but also ameliorated podocyte IR by activating the p-IRS1 (Tyr608)/p-PI3K/p-Akt signaling pathway. More importantly, this study also revealed the mechanism of ferroptosis in MGM attenuated renal injury in DN rats. MGM enhanced mevalonate-mediated antioxidant capacity (FSP1/CoQ10 axis and GPX4) and attenuated ACSL4-mediated proferroptotic lipid drivers’ generation in the kidney, thereby improving renal ferroptosis in DN rats (158).

### 3.2 Terpenoids

#### 3.2.1 Glycyrrhizic acid

Glycyrrhizic acid is a triterpene isolated from the traditional medicinal plant *Glycyrrhiza glabra*, which has various pharmacological properties such as anti-inflammatory, antibacterial, antioxidant, anti-fibrotic, antiviral, anti-diabetic, anti-obesity, anti-cancer, hepatoprotective, renoprotective, and immunomodulatory (159–162). Previous studies have demonstrated that glycyrrhizic acid is an effective inhibitor of HMGB1 and ROS production and that it can effectively attenuate renal injury in DN models (162, 163). Recent studies found that glycyrrhizic acid upregulated GPX4 expression and reduced ROS production in high glucose-induced mouse glomerular podocytes, suggesting that glycyrrhizic acid attenuates high glucose-induced podocyte injury in association with inhibition of ferroptosis (164).

#### 3.2.2 Ginkgolide B

Ginkgolide B is a diterpenoid isolated from the leaves of Ginkgo biloba with a variety of pharmacological effects, including anti-inflammatory (165), antioxidant (166), anti-apoptotic (167), and anti-ferroptosis (168, 169), etc. Recently, Chen et al. (170) found that ginkgolide B significantly reduced blood glucose in DN model mice, improved dyslipidemia and renal lipid accumulation, and attenuated renal dysfunction and histopathological damage, such as glomerular basement membrane thickening, tubular dilatation, extracellular matrix deposition, and fibrosis. Mechanistically, ginkgolide B promoted the expression of ferroptosis-associated proteins ferritin heavy chain 1 (FTH1) and GPX4, inhibited TfR1 expression, and decreased ROS levels and iron content in the DN model. Of these, inhibiting the ubiquitinated degradation of GPX4, increasing the level of GPX4 and thus inhibiting ferroptosis is the key of ginkgolide B to delay the progression of DN.

#### 3.2.3 Germacrone

Germacrone, a monocyclic sesquiterpene natural compound, is one of the main bioactive components of the Chinese medicine *Curcumaelongae Rhizoma*. Modern research has confirmed the significant anti-tumor (171), anti-viral (172), anti-fibrotic (173), anti-inflammatory (174), and antioxidant (174) effects of germacrone. The renoprotective effects of germacrone have also attracted the attention of researchers in recent years (175, 176). Jin et al. (177) found that germacrone reduced blood glucose and urinary protein in *db/db* mice and alleviated the structural damage to the kidney caused by hyperglycemia. Mechanistically, germacrone upregulated the expression of mmu_circRNA_0000309 in the DN model, followed by mmu_circRNA_0000309 sponge miR-188-3p which in turn promoted the expression of GPX4, thereby inhibiting ferroptosis and ultimately attenuating mitochondrial damage and podocyte apoptosis.

### 3.3 Anthraquinones

As we all know, Rhubarb and Senna are commonly used Chinese herbs for constipation. Sennoside A is an important bioactive component extracted from Rhubarb and Senna with a wide range of pharmacological activities, including laxative (178), anti-obesity (179, 180), anti-diabetic (181), anti-tumor (182), and hepatoprotective (183, 184). A recent study found that PTGS2 protein expression and MDA levels were significantly increased, while GPX4 protein expression and GSH activity were significantly decreased, in the kidneys of DN mice. Sennoside A treatment was able to reverse this phenomenon and reduce urinary protein levels. Mechanistically, sennoside A attenuated ferroptosis through inhibition of the Nrf2/HO-1 signaling pathway, thereby treating DN (185).

### 3.4 Alkaloids

#### 3.4.1 Berberine

Berberine, an isoquinoline alkaloid, is mainly extracted from the Chinese herb *Coptis chinensis*. Previous studies found that berberine has strong pharmacological activities, such as antidiabetic (186), hypolipidemic (187), antiviral (188), antitumor (189), anti-inflammatory (190), antioxidant (191), hepatoprotective (192), neuroprotective (193), etc. Currently, berberine is widely used in the treatment of diabetes and its complications, including type 2 diabetes (194), diabetic nephropathy (195), diabetic retinopathy (196), diabetic encephalopathy (196), and diabetic cardiomyopathy (197). Accumulating evidence suggests that berberine has a significant anti-ferroptosis effect (198–200). Bao et al. (200) found that berberine improved the viability and proliferation of pancreatic β cells. Mechanistically, berberine attenuated ferroptosis in β cells by promoting GPX4 expression. Guan et al. (201) demonstrated that berberine blocked high glucose-induced ferroptosis in glomerular podocytes by activating the Nrf2/HO-1/GPX4 signaling pathway, decreasing ROS levels, and increasing GSH activity.

#### 3.4.2 Leonurine

Leonurine is an alkaloid extracted from *Leonurus cardiaca* L. (motherwort), a plant in the Labiatae family, with various biological effects such as antioxidant (202–204), anti-inflammatory (204), regulating autophagy (205), improving mitochondrial dysfunction (203), and protecting pancreatic β cells (206). Huang et al. (207) thought that leonurine is a promising therapeutic agent to prevent diabetes and its complications by inhibiting the formation of advanced glycation end-products (AGEs). A recent study found that leonurine could reduce iron accumulation, lipid peroxidation, and ferroptosis by activating the Nrf2 antioxidant pathway (208). Similarly, Wu et al. (209) found that leonurine significantly reversed erastin-induced damage in HK-2 cells. Mechanistically, leonurine promoted p62 expression, activated Nrf2 entry into the nucleus, and upregulated HO-1 protein expression, thereby protecting HK-2 cells from ferroptosis injury. The above results suggest that leonurine may be a natural compound that effectively inhibits ferroptosis.

### 3.5 Coumarins

Umbelliferone is a natural coumarin compound extracted from Eucommiae Cortex, *Prunellae Spica*, and *Angelica Dahurica*. It has been reported to possess a variety of biological activities, including anti-diabetic and anti-hyperlipidemic (210), anti-diarrheal and anti-ulcerogenic (211), anti-rheumatic (212), anti-tumor (213), antivirulence (214), neuroprotective (215, 216), cardioprotective (217, 218), hepatoprotective (219), and renoprotective (220). Recent studies have found that umbelliferone shows great potential in DN treatment. Wang et al. (221) demonstrated that umbelliferone improved renal function in DN rats through modulation of inflammation and the TLR/NF-κB pathway. Jin et al. (222) observed that umbelliferone inhibited ROS production, increased GSH activity, decreased MDA levels and iron content, upregulated GPX4, Nrf2 and HO-1 expression, and downregulated ACSL4 expression in both *db/db* mice and high glucose-induced HK-2 cell models. This finding suggests that umbelliferone has inhibitory effects on oxidative stress and ferroptosis both *in vitro* and *in vivo*. However, this effect of umbelliferone was reversed when Nrf2 was knocked down. Thus, umbelliferone delays DN progression mainly by activating the Nrf-2/HO-1 pathway and inhibiting ferroptosis.

### 3.6 Lignans

Schisandrin A is the main bioactive component isolated from the traditional Chinese medicine *Schisandra chinensis*. Previous studies have shown that Schisandrin A has a variety of pharmacological effects, including anti-inflammatory, antioxidant, antitumor, antidiabetic, hepatoprotective, neuroprotective, and musculoskeletal protection (223). The emerging evidence has shown that Schisandrin A not only attenuated oxidative stress, the inflammatory response, mitochondrial damage, and ROS/TXNIP/NLRP3 signaling pathway-mediated pyroptosis in DN models but also inhibited high glucose-induced ferroptosis (224). Specifically, Schisandrin A reduced lipid ROS levels by activating AdipoR1/AMPK signaling, which in turn induced Nrf2, HO-1, GPX4, and SOD2 protein expression in the DN model. Therefore, Schisandrin A may be a potential therapeutic agent for DN.

### 3.7 Polysaccharides

In China, *Polygonatum sibiricum* is a medicinal and edible plant. *Polygonatum sibiricum* polysaccharides (PSP) are one of the main active ingredients of *Polygonatum sibiricum*, which is also the only content determination component of *Polygonatum sibiricum* prescribed by the Chinese Pharmacopoeia (2020 edition). Modern pharmacological studies have shown that PSP has antioxidant (225, 226), anti-aging (227, 228), anti-inflammatory (226), hepatoprotective (229), immunomodulatory (230, 231), and anti-diabetic (232) effects. A recent study has found that PSP significantly improved renal function parameters and attenuated renal histopathological damage in DN rats (233). Mechanistically, PSP reduced MAD and total iron content, increased GSH content, down-regulated transferrin and FTH1 expression, and up-regulated GPX4 expression in the renal tissues of DN rats. This study confirms that PSP can play a therapeutic role in DN by inhibiting ferroptosis.

### 3.8 Saponins

Platycodin D, a triterpenoid saponin, was isolated from the root of *Platycodon grandiflorum*. Previous studies have demonstrated that platycodin D has various pharmacological properties, such as anti-tumor (234, 235), anti-diabetic (236), anti-hypercholesterolemic (237), anti-inflammatory (238), anti-asthmatic (239), anti-obesity (240), and hepatoprotective effects (241). The emerging evidence has shown that platycodin D significantly reduced LDH release and increased cell viability in high glucose-induced HK-2 cells (242). Mechanistically, platycodin D inhibited lipid ROS production, decreased MDA levels and iron content, increased GSH levels, inhibited ACSL4 and TfR1 expression, and promoted FTH1, SLC7A11, and GPX4 expression. In addition, this effect was enhanced by the combination of platycodin D with the ferroptosis inhibitor deferasirox, while the effect was attenuated by GPX4 knockdown. Therefore, the reversal of high glucose-induced HK-2 cell injury by platycodin D was associated with upregulation of GPX4 expression and inhibition of ferroptosis.

### 3.9 Carotenoids

Crocetin, a natural carotenoid, is one of the main active ingredients in the traditional Chinese medicine saffron. Modern pharmacology has demonstrated that crocetin possesses a variety of pharmacological activities, including cholesterol-lowering (243), antidiabetic (244), antiatherosclerotic (245), antidepressant (246), cardioprotective (247), and nephroprotective (248, 249). In recent years, it has been found that crocetin could alleviate DN by inhibiting the expression of inflammatory cytokines and fibrotic factors in the kidney of diabetic rats (248). In high glucose-induced human glomerular mesangial cells, Lu et al. (250) observed that crocetin promoted Nrf2, GPX4, and HO-1 protein expression, reduced intracellular ROS production, and improved cell survival. Therefore, inhibition of ferroptosis in human glomerular mesangial cells is also an important mechanism for the prevention or treatment of DN by crocetin.

In conclusion, the active ingredients of some herbal medicines have shown good effects in the treatment of DN by targeting ferroptosis. Mechanistically, ferroptosis is inhibited mainly by enhancing antioxidant capacity, reducing lipid peroxidation levels, and reducing iron overload. Nrf2/HO-1 is a well-studied signaling pathway. GPX4 is a key signaling molecule for the antiferroptotic effect of Chinese herb medicine. Unlike other studies, Ding et al. (185) found that inhibition of excessive activation of the Nrf2/HO-1 signaling pathway was an important mechanism by which sennoside A attenuated ferroptosis in DN models. Therefore, scholars can explain this inconsistency in further studies.

## 4 Conclusion and prospects

As a newly discovered mode of cell death, ferroptosis has been a hot topic of research in the field of kidney disease in recent years. Throughout the past studies, great progress has been made in the study of ferroptosis in DN pathogenesis and in the prevention and treatment of DN through the regulation of ferroptosis by Chinese herbal medicine. From the available literature, multiple regulatory factors (non-coding RNAs, COX-2, ALOX15, Prdx6, ZIP14, HMGB1, Nrf2, and GPX4) and the HIF-1α/HO-1 pathway are involved in the regulation of ferroptosis in DN. A variety of herbal active ingredients such as glabridin, calycosin, glycyrrhizic acid, germacrone, berberine, etc. have been shown to reduce DN by targeting the inhibition of ferroptosis. However, the research on the regulation of ferroptosis in DN by Chinese herbal medicine is still in the preliminary exploration stage. There are still some limitations in the existing studies. Firstly, there is a lack of specific biomarkers for ferroptosis. Current studies can only indirectly demonstrate the occurrence of iron death by detecting ROS levels, lipid peroxidation products, iron levels, GPX4 activity, etc. Secondly, almost all studies are preclinical, and models are limited to cellular and animal models. Thirdly, most studies have only explored the molecular mechanism of Chinese herbal medicine to alleviate ferroptosis in DN *via* a single pathway. Finally, Chinese herbal medicine contains several components, such as Chinese herbal formulas, Chinese patent medicines, extractive compounds, etc. At the present stage, the research on Chinese herbal medicines is mostly limited to monomers and extractive compounds. Fewer studies have been conducted on Chinese herbal formulas and Chinese patent medicines that are used frequently in clinical practice. Therefore, in future research, we should focus on exploring the molecular mechanisms of ferroptosis regulation by Chinese herbal medicines from multiple pathways and perspectives, conducting relevant clinical trials, and further exploring new Chinese herbal medicines with anti-ferroptotic effects. We believe that as ferroptosis research progresses, it is expected to provide new effective therapeutic strategies for the treatment of DN.

## Author contributions

MW designed the study and completed the first draft, so she is the first author. XL examined the literature. ZT revised English grammar. XT and ML contributed in the scientific writing of the manuscript. MW and JW revised the manuscript. All authors contributed to the article and approved the submitted version.
